# Are We Heeding the Warning Signs? Examining Providers’ Overrides of Computerized Drug-Drug Interaction Alerts in Primary Care

**DOI:** 10.1371/journal.pone.0085071

**Published:** 2013-12-26

**Authors:** Sarah P. Slight, Diane L. Seger, Karen C. Nanji, Insook Cho, Nivethietha Maniam, Patricia C. Dykes, David W. Bates

**Affiliations:** 1 The Center for Patient Safety Research and Practice, Division of General Internal Medicine, Brigham and Women’s Hospital, Boston, Massachusetts, United States of America; 2 School of Medicine, Pharmacy and Health, The University of Durham, Stockton on Tees, Durham, United Kingdom; 3 Harvard Medical School, Boston, Massachusetts, United States of America; 4 Partners Healthcare Systems, Inc., Wellesley, Boston, Massachusetts, United States of America; 5 Department of Anesthesia, Critical Care and Pain Medicine, Massachusetts General Hospital, Boston, Massachusetts, United States of America; 6 Nursing Department, Inha University, Incheon, South Korea; Charité University Medicine Berlin, Germany

## Abstract

**Background:**

Health IT can play a major role in improving patient safety. Computerized physician order entry with decision support can alert providers to potential prescribing errors. However, too many alerts can result in providers ignoring and overriding clinically important ones.

**Objective:**

To evaluate the appropriateness of providers’ drug-drug interaction (DDI) alert overrides, the reasons why they chose to override these alerts, and what actions they took as a consequence of the alert.

**Design:**

A cross-sectional, observational study of DDI alerts generated over a three-year period between January 1st, 2009, and December 31st, 2011.

**Setting:**

Primary care practices affiliated with two Harvard teaching hospitals. The DDI alerts were screened to minimize the number of clinically unimportant warnings.

**Participants:**

A total of 24,849 DDI alerts were generated in the study period, with 40% accepted. The top 62 providers with the highest override rate were identified and eight overrides randomly selected for each (a total of 496 alert overrides for 438 patients, 3.3% of the sample).

**Results:**

Overall, 68.2% (338/496) of the DDI alert overrides were considered appropriate. Among inappropriate overrides, the therapeutic combinations put patients at increased risk of several specific conditions including: serotonin syndrome (21.5%, n=34), cardiotoxicity (16.5%, n=26), or sharp falls in blood pressure or significant hypotension (28.5%, n=45). A small number of drugs and DDIs accounted for a disproportionate share of alert overrides. Of the 121 appropriate alert overrides where the provider indicated they would “monitor as recommended”, a detailed chart review revealed that only 35.5% (n=43) actually did. Providers sometimes reported that patients had already taken interacting medications together (15.7%, n=78), despite no evidence to confirm this.

**Conclusions and Relevance:**

We found that providers continue to override important and useful alerts that are likely to cause serious patient injuries, even when relatively few false positive alerts are displayed.

## Introduction

Computerized physician order entry (CPOE) represents a valuable tool that allows medication and laboratory orders to be entered electronically by health care providers. While CPOE can substantially reduce the number of prescription-writing errors with even limited clinical decision support (CDS)[1], it is the CDS that makes it such a powerful application for improving patient safety in both inpatient and ambulatory settings; the impact will vary depending on the sophistication of the CDS[2]. CDS systems are designed to assist physicians in decision-making by providing them with real-time, relevant, patient-specific information and guidance at various stages in the health care process[3-6]. These systems can offer real practical benefits and substantial cost savings by alerting physicians to necessary drug-dosing adjustments, for example, based on a patient’s renal function, and potential hazardous drug-drug interactions (DDIs) and contraindications that need to be addressed[7,8]. 

However, CDS is variably successful—and the success of CDS systems often depend on both their intrinsic design and the knowledge bases that sit behind them. The Institute of Medicine has suggested that systems should be designed to make it “hard for people to do the wrong thing and easy for people to do the right thing”.[9] How closely the CDS advice matches a provider’s intentions, and how much control the provider has over assessing and responding to this, can influence its overall potential to improve patient safety[10]. Physicians are often inundated with irrelevant and inappropriate alerts, leading to high override rates. For example, Weingart et al. found that physicians overrode 91.2% of drug-allergy and 89.4% of high-severity drug interaction alerts in one study, when the threshold for alerting was set too low[11]. Too many alerts may result in ‘alert fatigue’, which can result in physicians overlooking even important clinically alerts[12]. Much of our recent work has focused on obtaining the right balance between useful alerting and over-alerting; an expert panel provided valuable guidance on those DDIs alerts that should be non-interruptive, as well as those contraindicated drug pairs for which physicians should always be alerted[13,14]. Interruptive alerts usually require the physician to provide a reason for their decision to override which infers an intention to carry out a particular action like, for example, will monitor drug levels as recommended. The problem with overriding these alerts is that it is often unclear whether, in fact, the provider has carried out this action or simply ignored it. In addition, how human factors issues are presented in the design of the alerts is also extremely important[15-17].

In this study, we evaluated how often and more importantly why providers overrode DDI alerts, in a setting in which relatively few false positive alerts were being delivered, as the knowledge base had already been “tuned” to address this issue[10]. Specific outcomes were: (1) the appropriateness of providers’ DDI alert overrides (2), the reasons why providers chose to override these alerts, and (3) what actions they took as a consequence of the alert. 

## Methods

### Research Study site

This study included 36 primary care practices affiliated with two Harvard teaching hospitals, Brigham and Women’s Hospital and Massachusetts General Hospital (Boston, MA). A total of 1718 prescribers serve these sites, which are part of a regional integrated healthcare delivery system - Partners HealthCare.

### LMR and Clinical Decision Support

Partners HealthCare physicians working in the ambulatory setting use a self-developed, Certification Commission for Healthcare Information Technology (CCHIT)-approved, electronic health record (EHR) - the Longitudinal Medical Record (LMR). Implemented in 2000, the LMR allows providers to document patient problems, medications, allergies, and encounter notes; access laboratory and radiology reports; write prescriptions and communicate with other healthcare providers. The LMR has clinical decision support capability in the form of medication alerts for drug-allergy and DDIs, and drug suggestions in patients with renal failure, geriatrics, and those on duplicate therapy. DDI alerts are generated at the time of ordering, and use data from patients’ active medication list and existing Partners DDI knowledge base. Partners DDI knowledge base currently consists of approximately 5,000 active DDI rules, sourced from commercial knowledge bases such as First DataBank, Inc., and reviewed and approved by the Partners DDI Content Committee. This knowledge base has been iteratively improved over time to reduce the number of false positive alerts[10,13,14].

**Figure 1:**
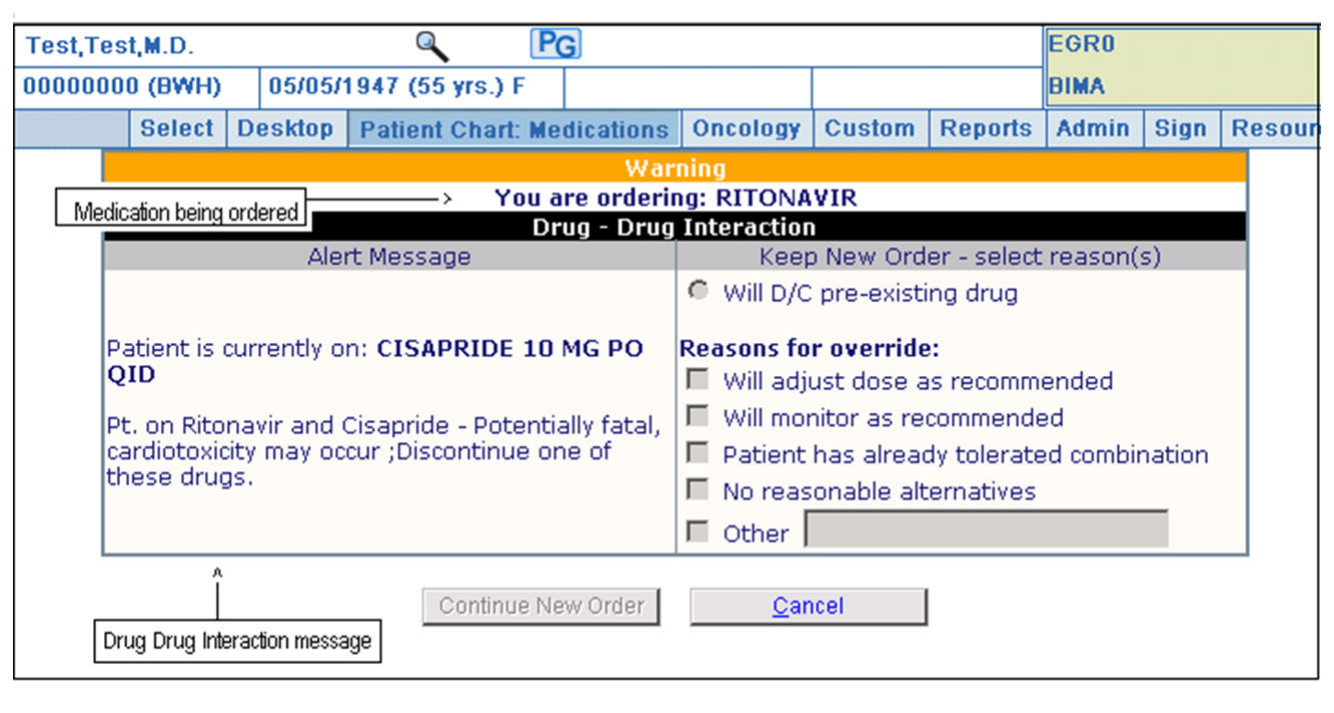
Screenshot of a Level 2 alert.

When a DDI alert is generated, a specific recommendation is presented to the physician, which is linked to a monograph. The recommendation includes the specific type of interaction and often suggests a particular course of action (*e.g., avoid concurrent use of both drugs*). Level 1 alerts indicate a very serious, life-threatening interaction and require the provider to discontinue the interacting medicine in order to proceed. Level 2 alerts suggest an undesirable interaction likely to cause serious injury, and give the provider the option of ‘cancelling’ the order or ‘overriding’ the alert. Should the physician choose to override the alert, they are required to select one of the following coded reasons in order to proceed: “Will monitor as recommended”, “Will adjust dose as recommended”, “Patient has already tolerated combination”, “No reasonable alternatives” or “*Other*” (Figure **1**). If “*Other*” is selected, further details should be entered in the free-text field. The provider will also be given the opportunity to order follow-up lab tests at this time, if indicated. Level 3 alerts specify the possibility of a less serious, undesirable interaction and are presented as non-interruptive alerts. 

### Study design and sample selection

This study was a cross-sectional, observational study of DDI alerts generated over a three-year period between January 1st, 2009, and December 31st, 2011. Access to these data required approval of the Partners Human Research Committee (PHRC), which is the Institutional Review Board (IRB) of Partners Research Management at Partners HealthCare. On receiving specific IRB approval for this study, all Level 2 DDI alerts that were overridden were downloaded (total 14,966 overrides, 60.2% of alerts generated). Patients who did not give permission for their information to be stored in the hospital database and used for research were excluded. Our sample was then limited to providers who had received 20 or more DDI alerts (opportunity to override), and the number of times each provider overrode these alerts calculated. The top 62 providers with the highest override rate were identified and eight overrides randomly selected for each provider (a total of 496 alert overrides for 438 patients, 3.3% of the sample). The downloaded file included patients’ names and medical record identification numbers; names of both medicines that triggered the DDI alert; date of alert; practice location; prescribers’ names, identification numbers, sex, age, level and experience; and the reasons given by providers at the time of overriding the alert. Any duplicates were removed and replaced, and patient information anonymized prior to analysis. 

In the initial analysis, a physician and pharmacist expert panel screened the sample for severe or contraindicated interactions, based on the strength of the supporting clinical evidence and the severity of the adverse events in different knowledge bases, such as Partners Healthcare System Medication Knowledge Base (PHS MKB); commercial medication knowledge bases such as Micromedex (New York, New York, USA); First DataBank (FDB) (San Francisco, California, USA); and http://www.drugs.com. Alert overrides of therapeutic duplications (e.g., sumatriptan and rizatriptan) and therapeutic combinations that put the patient at an increased risk of (i) serotonin syndrome (e.g., fluoxetine-sumatriptan), (ii) seizures (e.g., tramadol-cyclobenzaprine), (iii) infection (e.g., hydroxyurea-zoster vaccine live), (iv) bone marrow suppression (e.g., methotrexate-trimethoprim), (v) bleeding (e.g., dabigatran-ketoconazole), (vi) cardiotoxicity (e.g., QT prolongation, torsades de pointes, cardiac arrest), (vii) opioid withdraw symptoms (e.g., morphine-naltrexone), (viii) priapism (e.g., tadalafil-clarithromycin), (ix) sharp falls in blood pressure (e.g., sildenafil-nitroglycerin), or significant hypotension (e.g., sildenafil-tamsulosin), (x) reduced virologic response (e.g., tenofovir-atazanavir), and (xi) myopathy / rhabdomyolysis (e.g., diltiazem-high dose simvastatin) were all considered inappropriate. Therapeutic combinations that may result in decreased bioavailability and clinical effectiveness of one or both drugs were also considered inappropriate. If the provider indicated that there were “no reasonable alternatives”, “the patient had already tolerated the combination”, or they had “*other*” reason(s) for prescribing both drugs together, the record was put forward for detailed chart review. If the provider gave more than one reason, the carrying out of both actions was assessed. Topically applied, ophthalmic and otic preparations were considered appropriate. Alert overrides of DDIs involving epinephrine autoinjector were also considered appropriate when prescribed for severe allergic reactions. 

### Detailed chart review

The purpose of the review was to ascertain whether the provider had carried out their intended action(s). Table **1** and **2** contain the criteria used for assessment of all appropriate alert overrides. An academic pharmacist (S.P.S) reviewed the electronic medical record for each of the 338 overrides from the date the alert was triggered. An attending physician (K.C.N.) independently reviewed a sample of these medical charts (23.7%, n= 80) and inter-rater agreement calculated and found to be excellent (κ=0.84). Any disagreements were resolved by discussion with a third reviewer (D.W.B.). 

**Table 1:** Criteria for assessment of intended actions.

Coded Reason for DDI Alert Override	Criteria
1. Will monitor as recommended	Test ordered within the specified time period (see Table *2*)
2. Patient has already tolerated combination	Five half-lives of the newly started or more recently started medicine have elapsed
3. No reasonable alternative	Evidence suggests that no other drug within the same therapeutic class was a safer alternative, and/or appropriate monitoring/dose adjustment was conducted
4. Will adjust dose as recommended	The dose was adjusted according to the alert recommendations, or If no recommended dose was specified, the appropriate monitoring was carried out and any necessary dose adjustment made during the course of treatment.

**Table 2:** Time period within which the test should be ordered after alert override.

	Time period
Creatine kinase	12 weeks
Cyclosporine	8 weeks
Digoxin	2 weeks
HbA1c	12 weeks
Lithium	12 weeks
Methotrexate	3 days
PT-INR (*Prothrombin Time - International Normalized Ratio*)	3 weeks
Respiratory status	4 weeks
Sirolimus	2 weeks
Tacrolimus	1 week

### Data Analysis

Data were downloaded directly into Microsoft Excel 2011 (Microsoft Corp, Redmond, WA). Descriptive statistics were used to summarize the alert overrides considered appropriate, the drugs and DDIs responsible for generating the majority of alerts, the override reasons given by providers and the actions they took. Comparisons involving categorical variables were performed using Rao-Scott chi-square test statistics, adjusting for clustering of providers. We used SAS statistical software, version 9.3 (SAS Institute Inc., Cary, NC, USA) for all statistical analyses.

## Results

### Summary

After the initial screening, 68.2% (338/496) of the DDI alert overrides were considered appropriate. A detailed review of the medical charts revealed that the desired action was only carried out in 63.3% (214/338) of these cases. One hundred and thirteen different drugs, and 119 different drug-drug interactions, were found to have triggered the 496 DDI alerts. Eight drugs in particular were responsible for generating approximately three quarters of these alerts: simvastatin (20.6%, n=102), sildenafil (9.9%, n=49), tramadol (9.5%, n=47), citalopram (7.7%, n=38), amlodipine (7.5%, n=37), tamsulosin (7.1%, n=35), azithromycin (6.5%, n=32), and warfarin (6.3%, n=31). 

### Drug-drug and class-class interactions

Cumulatively, 44 of the different drug-drug interactions accounted for over half (265/496) of the alerts shown to providers, and could be categorized into ten class-class interactions (Table **3**). The calcium channel blockers – statins (class-class) interaction was triggered most often (14.5%, n=72), with amlodipine – simvastatin (drug-drug) interaction accounting for over half (51.3%, n=37) of the alerts. The phosphodiesterase type-5 inhibitors – alpha-adrenoceptor blocking drugs (class-class) interaction was second highest (10.7%, n=53), with sildenafil – tamsulosin (drug-drug) interaction making up less than half (47.2%, n=25) of the alerts generated. The selective serotonin reuptake inhibitors – 5 HT_1_ receptor agonists (class-class) interaction and selective serotonin reuptake inhibitors – opioid analgesics (class-class) interaction were third (7.3%, n=36) and fourth highest (3.4%, n=17) respectively. In the latter group, the citalopram – tramadol (drug-drug) interaction triggering over half of these alerts (58.8%, n=10). 

**Table 3:** Top 10 drug class-class interactions that were overridden.

	**Object Drug / Class** *	**Precipitant Drug / Class** ^†^	**Total no. (%) of alert overrides**
1.	Calcium channel blockers (*i.e., amlodipine; diltiazem; verapamil*)	Statins (*i.e., simvastatin; lovastatin*)	72 (14.5)
2.	Phosphodiesterase type-5 inhibitors (*i.e., sildenafil; tadalafil; vardenafil*)	Alpha-adrenoceptor blocking drugs (*i.e., tamsulosin; terazosin; doxazosin; alfuzosin*)	53 (10.7)
3.	Antidepressants - selective serotonin reuptake inhibitors (*i.e., citalopram; sertraline; fluoxetine; paroxetine; escitalopram; fluvoxamine*)	5 HT_1_ receptor agonists - ‘Triptans’ (*i.e., sumatriptan; eletriptan; zolmitriptan; rizatriptan; almotriptan*)	36 (7.3)
4.	Antidepressants - selective serotonin reuptake inhibitors (*i.e., citalopram; sertraline*)	Opioid Analgesics (*i.e., tramadol*)	17 (3.4)
5.	Antidepressants – tricyclic (*i.e., amitriptyline; nortriptyline, doxepin, imipramine*)	Opioid Analgesics (*i.e., tramadol*)	16 (3.2)
6.	Antibacterial drugs – macrolides (*i.e., azithromycin; clarithromycin*)	Statins (*i.e., simvastatin; atorvastatin*)	16 (3.2)
7.	Central nervous system stimulants (*i.e., amphetamine / dextroamphetamine*)	Proton pump inhibitors (*i.e., omeprazole; pantoprazole*)	15 (3.0)
8.	Antibacterial drugs – sulphonamides and trimethoprim (*i.e., trimethoprim/sulfamethoxazole*)	Oral anticoagulants – coumarins (*i.e., warfarin*)	14 (2.8)
9.	Lipid-regulating drugs – fibrates (*i.e., gemfibrozil*)	Statins (*i.e., simvastatin; rosuvastatin*)	13 (2.6)
10.	Sympathomimetics (e.g., epinephrine autoinjector)	Beta-adrenoceptor blocking drugs (*i.e., propranolol; labetalol*)	13 (2.6)
	Total		265 (53.4)

^*^ The object drug was defined as the drug that has its therapeutic effect modified by the interaction process.

^†^ The precipitant drug was defined as the drug responsible for affecting the pharmacologic action or the pharmacokinetic properties of the object drug.

### Appropriateness of alert overrides

Over one third (158/496) of alert overrides were judged to be inappropriate as the therapeutic combinations put patients at increased risk of (i) serotonin syndrome (21.5%, n=34), seizures (4.4%, n=7), or both (5.7%, n= 9); (ii) infection (2.5%, n=4); (iii) bone marrow suppression (1.9%, n=3); (iv) bleeding (0.6%, n=1), (v) cardiotoxicity (16.5%, n=26); (vi) opioid withdraw symptoms (0.6%, n=1), (vii) priapism (8.2%, n=13), (viii) sharp falls in blood pressure or significant hypotension (28.5%, n=45); or (x) reduced virologic response (1.3%, n=2). Therapeutic duplications (1.9%, n=3) and therapeutic combinations (6.3%, n=10) that may result in decreased bioavailability and clinical effectiveness of one or both drugs were also considered inappropriate. Over half of the providers (59.7%, n=37) were found to have inappropriately overridden three or more alerts. 

### Reasons for alert overrides

The most common coded reasons for overriding DDI alerts were ‘will monitor as recommended’ (43.9%, n=218), ‘will adjust dose as recommended’ (16.9%, n=84), and ‘patient has already tolerated combination’ (15.7%, n=78). Providers chose the coded reason ‘other’ in 19.7% (n=98) of alert overrides, providing a free-text explanation for why they chose to override the alert in only 3.6% (n=18) of cases (Table **4**). In 13 of these 18 cases, the provider commented that the drug had been recommended by another healthcare provider (n=6), the patient had already been counseled not to take both drugs together (n=3), or the patient was no longer taking one of the drugs listed as a potential cause of the interaction (n=4). In two more cases, the provider wrote that they were tapering one of the interacting drugs. 

**Table 4:** Coded reasons given by providers for overriding DDI alerts.

**Coded Reasons**	**No.** (**%**)*
Will monitor as recommended	218 (43.9)
Will adjust dose as recommended	84 (16.9)
Patient has already tolerated combination	78 (15.7)
No reasonable alternatives	2 (0.4)
Other (with no free text reason provided)	80 (16.1)
Other (with free text reason provided)	18 (3.6)
Combinations of the coded reasons listed above	16 (3.2)
**Total**	496

^*^ Percentages have been rounded and may not total 100.

### Carrying out the desired actions

The desired action was carried out in only 63.3% (214/338) of cases. Of the 121 appropriate alert overrides where the provider said they would “monitor as recommended”, a detailed chart review revealed that only 35.5% (n=43) actually completed the monitoring. Where the provider indicated that they would “adjust dose as recommended”, 60% (n=21) of 35 appropriate alert overrides adjusted the dose according to the alert recommendations; if no recommended dose was specified, the appropriate monitoring was found to have been carried out and any necessary dose adjustment made during the course of treatment. Where the provider selected the coded reason “patient has already tolerated combination”, five half-lives of the newly started (or more recently started) medicine had elapsed in 80.8% (n=63) of cases. Providers chose the coded reason “other” (without providing a free-text explanation) in 16.1% (n=80) of alert overrides; a potential reason was found in the notes for 87.5% (n=70) of these cases, with patients previously prescribed both drugs together in 58.8% (n=47) of cases. 

### Providers’ attributes associated with alert overrides

We conducted a univariable analysis to see if factors such as sex, age, and level and experience, were each associated with the decision to 1) appropriately override the alert and 2) carry out the intended action (Table **5**). Providers’ level and experience appeared to be the only factor associated with the decision to carry out the intended action, with staff physicians more experienced and less likely to carry out the intending action than to carry them out (61.3% vs 31.1%; p<.001). 

**Table 5:** Providers’ attributes associated with alert overrides.

	Stage 1: Initial screening				Stage 2: Chart review			
	No. (%) of alert overrides*				No. (%) of alert overrides*			
Provider attributes	Appropriate (n=338)	Inappropriate (n=158)	Total (n=496)	P Value	Actioned (n = 214)	Not actioned (n = 124)	Total (n=338)	P Value
Sex				0.0022				0.1837
Male (n=25)	112(33.1)	88(55.7)	200(40.3)		63(29.4)	49(39.5)	112(33.1)	
Female (n=37)	226(66.9)	70(44.3)	296(59.7)		151(70.6)	75(60.5)	226(66.9)	
Age, y				0.6151				0.1786
< 35 (n=14)	77(22.8)	35(22.2)	112(22.6)		46(21.5)	31(25.0)	77(22.8)	
35 - 65 (n=45)	249(73.7)	111(70.3)	360(72.6)		163(76.2)	86(69.4)	249(73.7)	
> 65 (n=3)	12(3.6)	12(7.6)	24(4.8)		5(2.3)	7(5.7)	12(3.6)	
Level & experience				0.1019				<.0001
Staff physician (n=31)	143(42.3)	105(66.4)	248(50.0)		67(31.3)	76(61.3)	143(42.3)	
House officer/fellow (n=4)	25(7.8)	7(4.4)	32(6.5)		16(7.5)	9(7.3)	25(7.4)	
Nurse (n=9)	59(17.5)	13(8.2)	72(14.5)		49(22.9)	10(8.1)	59(17.5)	
Medical assistants (n=5)	34(10.1)	6(3.8)	40(8.1)		33(15.4)	1(0.8)	34(10.1)	
Resident (n=7)	39(11.5)	17(10.8)	56(11.3)		21(9.8)	18(14.5)	39(11.5)	
Unknown/Undisclosed (n=6)	38(11.3)	10(6.3)	48(9.7)		28(13.1)	10(8.1)	38(11.2)	

^*^ Percentages have been rounded and may not total 100.

## Discussion

Computer order entry linked with CDS holds great promise for improving medication safety, quality, and efficiency. However, many implementations have not achieved the desired results, and DDIs have been an especially complex domain. Few studies have looked at the appropriateness of DDI alert overrides or whether providers actually carried out their intended actions. In this study, providers appropriately overrode just over two-thirds of the DDI alerts and carried out the intended action in less than two-thirds of these cases. 

We found that just eight drugs were responsible for generating approximately three quarters of important DDI alerts in this population. Drawing comparisons with existing literature, Weingart et al.[11] found that certain drugs, one of which was similar (e.g., azithromycin), accounted for approximately one third of alerts. Taking a larger sample of drug interaction alerts, we found different interactions generated over half of the alert overrides. Some of these combinations should essentially never be used as they put patients at increased risk of potential lethal arrhythmia (torsades de pointes). Despite extensively modifying our CDS system with fewer but more meaningful interruptive alerts[10], our study shows that providers continue to override important and useful alerts, which are likely to cause serious patient injuries (Level 2). This raises broader questions around whether we should be reaching out to providers who are not prescribing optimally and working with them to improve their prescribing.

To understand why users chose to override these alerts, this study also captured the coded and free-text reasons they provided. Similar to a previous study[10], the majority of providers chose the coded reason “will monitor as recommended”, with others selecting the coded reason “other” and then leaving the free-text box blank. Failure to provide free-text explanations for why alerts were overridden may conceal good clinical reasoning. A key difference between our study and the Shah et al. study [10] is that we also reviewed the charts to see whether the provider actually took action (e.g., monitored the required levels or adjusted the dose) as a consequence of the alert. Our study showed how the intended action was carried out in only two-thirds of cases. This is an important finding as it raises concerns over patient safety. Failure to monitor a patient’s drug levels (e.g., digoxin) after initiation of an interacting drug (e.g., verapamil) can result in potentially serious side effects for the patient[18]. Our study also revealed how some providers selected the coded reason “patient has already tolerated combination”, yet no information was found to suggest that the patient had been taking both drugs together previously. Although difficult to say for certain, it is possible that some providers may have randomly chosen this (or indeed potentially any) coded reason in order to proceed with the order. More research is needed to explore the human factors elements that influence provider behaviour, including non-clinical motivations of providers, such as patient demands, workload, time constraints in a busy office practice, attitudes towards particular diseases or patients, habits, and peer influence. 

Like any research study, this study had inherent limitations. It was undertaken within a single healthcare delivery system using one outpatient prescribing system and, as such, is difficult to assess how generalizable the results are to other prescribing systems. It was also possible for patients to have been receiving care from providers outside the integrated Partners HealthCare system. In some cases, this may not have been documented in the patients’ records. Notwithstanding these limitations, the findings have important implications for the future design of clinical decision support alerts. 

## Conclusion

An important aspect of CDS is screening of severe DDIs and prompting providers to take action to prevent concomitant use. We found that, despite extensively modifying our CDS system to improve user acceptance and show only the most important alerts, providers continued to override these alerts that are likely to cause serious patient injuries. More needs to be done to effectively feedback to providers with high inappropriate override rates and improve prescribing safety in the primary care setting. 
